# Associations of Fuchs heterochromic iridocyclitis in a South Indian patient population

**DOI:** 10.1186/1869-5760-3-14

**Published:** 2013-01-15

**Authors:** Kalpana Babu, Madhura Adiga, Sunil R Govekar, BV Ravi Kumar, Krishna R Murthy

**Affiliations:** 1 Department of Uveitis & Intraocular Inflammation, Vittala International Institute of Ophthalmology, Bangalore, 560085, India; 2 Department of Uveitis & Intraocular Inflammation, Prabha Eye Clinic and Research Center, 504, 40th Cross, Jayanagar 8th Block, Bangalore, 560070, India; 3 Molecular Diagnostics laboratory, XCyton, Bangalore, 560058, India

## Abstract

**Background:**

The purpose of this study is to look for any possible associations in 58 consecutive cases of Fuchs heterochromic iridocyclitis (FHI) in a South Indian patient population. Fifty-eight consecutive cases (59 eyes) of FHI underwent a detailed ocular and systemic evaluation. Routine laboratory investigations for uveitis including serum angiotensin-converting enzyme and enzyme-linked immunosorbent assay (ELISA) for toxoplasmosis (IgG and IgM) were done in all the cases. Syndrome Evaluation System comprising of multiplex nucleic acid amplification and signature specific hybridization on the aqueous fluid was done in all 59 eyes for herpes simplex virus (HSV), varicella zoster virus, cytomegalovirus (CMV), rubella virus, chikungunya virus, *Toxoplasma*, and *Mycobacterium tuberculosis*. The results were statistically assessed using the SPSS (version 15) package.

**Results:**

Thirty-three males and 25 females with FHI were included in the study. Systemic sarcoidosis was seen in two cases. Serological tests failed to confirm an association with toxoplasmosis in all the cases. Aqueous fluid analysis showed positivity only to HSV (one case), CMV (one case), and chikungunya virus (one case).

**Conclusions:**

We do see associations of sarcoidosis, HSV, and CMV in FHI in our patient populations as well. The detection of chikungunya virus in a patient with FHI in our series adds to the list of associations with FHI.

## Background

Fuchs heterochromic iridocyclitis (FHI) is a disease of unknown etiology characterized by low-grade intraocular inflammation, iris heterochromia or atrophy or both, characteristic keratic precipitates (KPs) distributed all over the endothelium, absence of synechiae, development of cataract, and less frequently of glaucoma [1,2]. Previous associations with toxoplasmosis [3], toxocariasis [4,5], sarcoidosis [6], rubella vaccination [7], cytomegalovirus [8,9], herpes simplex virus [10], chikungunya virus infections [11,12], retinitis pigmentosa [13], Horner’s syndrome [14], Usher’s syndrome, and previous trauma [15] have been described in the literature. The purpose of this study is to look at any possible known associations in consecutive cases of FHI in a South Indian patient population.

## Results

Fifty-eight patients (33 males and 25 females, 59 eyes) with FHI were included in the study. A unilateral involvement was noted in 57 cases (98.30%). The right eye was involved most commonly in 31 eyes (52.54%). Blurring of vision was the most common presentation seen in all cases (100%). The best corrected visual acuity (BCVA) on presentation was 6/12 or better in 48 eyes, 6/18 to 6/60 in 8 eyes, and <6/60 in 3 eyes. Stellate and medium-sized KPs were seen in all patients. Pigmented KPs were seen in nine eyes (15.25%). Most common location of the KPs (57 eyes) was all over the endothelium (96.61%). The remaining three eyes had the KPs located central and inferiorly. Mild anterior chamber reaction was seen in all cases (100%). Iris pattern changes included varying degrees of depigmentation including moth-eaten appearance in 41 eyes (69.49%), heterochromia in 17 eyes (28.81%), and abnormal atrophic areas in the iris stroma in 1 eye (1.7%). Koeppe nodules were seen at the pupillary margin in five eyes (8.48%). Busacca nodules were seen in one eye. Complicated cataract was seen in 22 eyes (37.28%). Pseudophakia was noted in ten eyes (16.95%). Well-functioning bleb was noted in two eyes with a history of trabeculectomy. Vitreous opacities were noted in 19 eyes (32.2%). Few small, multiple chorioretinal atrophic areas were seen in the retinal periphery in one eye (1.69%). All patients had a good foveal reflex, and none had disc hyperemia. Intraocular pressure (IOP) was raised in ten eyes (16.95%); three out of the ten eyes had an increase in IOP between 41 to 50 mmHg. Glaucomatous changes in the optic nerve head were seen in two eyes.

**Figure 1:**
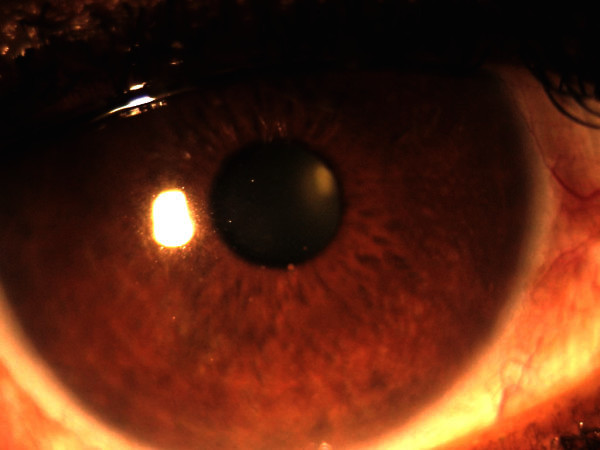
**Slit lamp photograph of the right eye of patient with HSV positivity.** Showing areas of altered iris pattern in diffuse illumination (**A**) and pigmented keratic precipitates (**B**) in slit illumination.

Serum angiotensin-converting enzyme was increased in two cases. Chest X-ray showed mediastinal lymphadenopathy in two cases. Biopsy diagnosis of sarcoidosis was made in one case, while in the second case, the diagnosis of sarcoid was made on the basis of the ancillary laboratory investigations. Serological tests failed to confirm an association with toxoplasmosis in all the cases. Syndrome evaluation system (SES) on the aqueous tap was positive for herpes simplex virus (HSV) in one case, cytomegalovirus (CMV) in one case, and chikungunya virus in one case. The aqueous tap was negative in all cases for varicella zoster virus (VZV), rubella virus, *Toxoplasma*, and *Mycobacterium tuberculosis*. The eye that tested positive for HSV (Figure 1) had a history of recurrent inflammation, increase in IOP, pigmented KPs, and irregular iris stromal atrophic areas. He was treated with oral acyclovir 400 mg five times per day for a week followed by two times per day for a month along with nepafenac eye drops, timolol 0.5% eye drops bid, and dorozolamide eye drops tid.

**Figure 2:**
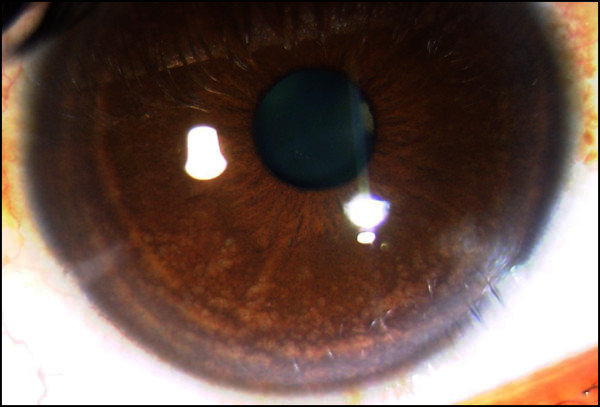
**Slit lamp photograph of the right eye of patient with CMV positivity.** Showing the moth-eaten iris pattern (**A**) and prominent central keratic precipitates (**B**) in diffuse illumination.

The eye that tested positive for CMV did so on three occasions. She was initially diagnosed to have FHI with secondary glaucoma and was on and off on treatment with dexamethasone eye drops for her inflammations. She had the classical moth-eaten appearance of the iris with no posterior synechiae and keratic precipitates located centrally (Figure 2). She had an IOP of 42 mmHg, which continue to persist with maximum antiglaucoma medications. Aqueous tap was positive for CMV. Her IOPs and inflammation did settle temporarily with oral valganciclovir, fluoromethalone eye drops, and ganciclovir eye ointment. During one of the follow-ups, she consulted a glaucoma specialist at a different center, who unknowingly started her on prostaglandin analogues. Within 2 days, she developed a reactivation of anterior uveitis with increase in IOP. She underwent trabeculectomy. Her aqueous sample was again positive for CMV. At 2 years follow-up, her eye is free of inflammation and the IOP has been stable.

The eye positive for chikungunya virus had pigmented KPs distributed all over the endothelium, moth-eaten appearance of the iris, cataract, and an IOP of 30 mmHg. Aqueous tap was positive for chikungunya virus. Retrospectively, a history of chikungunya fever 1.5 months ago was elicited from the patient. These details were not revealed by the patient during the initial examinations.

## Discussion

**Figure 3:**
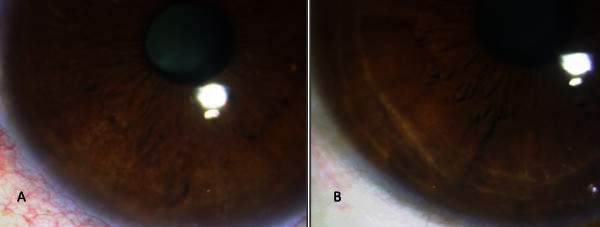
**Slit lamp photograph.** Showing Fuchs heterochromic iridocyclitis (**A**) and the normal other eye (**B**) in diffuse illumination.

Ernst first described FHI in 1906 [1]. Since then, many theories have been proposed regarding its pathogenesis (stimulus being immunological, infection or both) [2]. FHI is thus probably a secondary phenomenon with a spectrum of clinical signs and multiple causes. The diagnosis of FHI to date is made on clinical grounds (Figure 3) with no diagnostic test available. In an attempt to look at any possible disease associations in our patient population, this study with laboratory investigations and aqueous fluid analysis on 59 consecutive eyes of FHI was done.

The mean age in our series was 37.95 ± 12.42 years (range 16 to 68 years). Unilateral involvement was the most common presentation. Bilateral involvement is rare in our population. This is comparable to the existing literature on FHI [16].

The most common complaint at presentation was blurred vision (100%). Good visual acuity at presentation was seen in most of our patients. The most common causes of decrease in visual acuity were cataract (22 eyes) and glaucomatous changes in the optic nerve head (2 eyes). The visual prognosis in FHI was good especially in those eyes with normal IOP.

The keratic precipitates in FHI have been classically described as having a stellate morphology with fibrillary extensions [1,2]. Medium-sized, round, non-confluent KPs were the most common presentation in our series. Pigmentation is usually rare in FHI. In our series, we had nine eyes that had pigmented KPs. Out of these nine eyes, six eyes had increased IOP at the time of inflammation. Among these six eyes, three eyes documented positive virus nucleic acids of CMV (one eye), HSV (one eye), and chikungunya virus (one eye) The most common distribution of KPs was all over the endothelium in 56 eyes. Central and inferior location of KPs was seen in three eyes. These three eyes also presented with increased IOP. One of these eyes tested positive for CMV.

Variable amount of iris stromal atrophy was seen in all our patients. Moth-eaten appearance of the iris was the most common iris stromal change seen in our series. Koeppe nodules were the most common iris nodules seen. Busacca nodules were seen in only one eye. This is comparable to the existing literature on FHI [16].

Vitreous debris and opacities were seen in 19 eyes. In all these eyes, fundus evaluation showed a good foveal reflex with no disc hyperemia. One patient had small chorioretinal scars in the periphery, which could be due to her myopia. None of the eyes in our series had toxoplasmosis related chorioretinal scars and negative serologic testing for toxoplasmosis antibodies. Ganesh et al. have reported FHI in a single patient with bilateral ocular toxoplasmosis from the Indian subcontinent [3].

Increase in IOP was seen in ten eyes. A virus was detected in three eyes only. The cause of increase in IOP in the remaining seven eyes at the time of inflammation could not be found.

On review of the laboratory investigations in all our cases, sarcoidosis was seen in two cases. Whether this association is a coincidence is a speculation. Goble and Murray [6] have described a case series of association of FHI with sarcoidosis.

The aqueous fluid of all patients in this study were tested by SES [17] for the presence of nucleic acids of HSV, VZV, CMV, rubella virus, chikungunya virus, *Toxoplasma*, and *Mycobacterium tuberculosis* (MTb) simultaneously in a single sample and in a single test. This is the highlight of this paper. Controls included aqueous samples from 25 patients (20 cases undergoing routine cataract surgery and 5 cases of Vogt-Koyanagi Harada disease). The microbiologists performing the PCR were masked to the diagnosis. Out of 59 eyes with FHI, 3 eyes tested positive for virus (HSV, CMV, and chikungunya virus). All the control aqueous samples were negative for the above organisms.

An association of HSV with FHI has been described earlier by Barequet et al. [10]. In our series, we had only one eye with HSV. Associations of CMV with FHI have been described recently in the literature [8,9]. We had one eye with CMV positivity in FHI in our series, which implies that CMV is seen in our patient population as well. CMV-associated inflammations are usually associated with high spikes in IOP and it may be worthwhile to do an aqueous fluid analysis in all patients with FHI with increased IOP. It is also important to rule out a viral etiology in FHI, as newer antiglaucoma medications like prostaglandin analogues can reactivate a latent virus in the corneal endothelial cells. There have been isolated reports on the detection of chikungunya virus in FHI in the literature. Due to the high viraemias present during chikungunya fever, there could be spilling of the chikungunya virus into the body fluids [12]. Our patient had a chikungunya fever 1.5 months before he presented with ocular manifestations. As we were seeing this patient for the first time during this study, without any records of previous ocular evaluations, it is difficult to speculate the association of FHI and chikungunya virus in this patient. Had the patient been followed up by serial testing of aqueous fluid, a definitive correlation between the presence of the virus with clinical syndrome could have been established. Nevertheless this virus has also been detected in a patient with FHI during aqueous fluid analysis.

Although rubella virus has been described in the etiology of FHI, we did not detect the rubella virus in any of our patients. Antigen-antibody complexes [15] in the aqueous sample were not done in any of our patients due to financial constraints. This is one of the limitations of this study and would have probably given us more comprehensive information regarding the association of rubella virus in our patients with FHI, especially in those eyes presenting with increased IOP.

## Conclusions

We do see associations of sarcoidosis, HSV, and CMV with FHI in our patient populations as well. The detection of chikungunya virus in a patient with FHI in our series along with other isolated reports from India adds to the list of associations with FHI.

## Methods

Fifty eight consecutive cases of FHI underwent a detailed ocular and systemic evaluation. The diagnosis of FHI was made on clinical observation of low-grade intraocular inflammation, iris heterochromia, atrophy or both, characteristic KPs, absence of posterior synechiae and development of cataract, and less frequently of glaucoma. The following information was retrieved from all the 58 cases: age, gender, laterality, type of presentation (acute, chronic, acute on chronic), any significant medical or surgical history, and any associated systemic disease. Details of ocular evaluation included the eye involved, BCVA, morphology (fine, medium, large, and pigmented) and distribution of KPs, anterior chamber reaction (cells and flare), iris nodules, pattern of iris atrophy, heterochromia, size of the pupil, presence of cataract, vitreous reaction (haze and cells), any disc hyperemia, optic nerve changes, chorioretinal scars, or peripheral retinal involvement. Intraocular pressure (Goldman aplannation tonometry) was recorded in all patients. Details of systemic evaluation included history and review of all systems. Laboratory investigations included a complete hemogram with the erythrocyte sedimentation rate, C-reactive protein, serum angiotensin-converting enzyme, enzyme-linked immunosorbent assay (ELISA) from the serum tested for toxoplasmosis antibodies (IgG and IgM), VDRL, Mantoux test, rheumatoid factor, antinuclear antibody, and HLAB27. Chest X-ray was done in all the patients. Additional CT scan was done in one patient. ELISA on serum for cytomegalovirus antibodies (IgG and IgM) was done in one patient. All patients underwent an aqueous tap from the involved eye, and the aqueous sample was sent for syndrome evaluation system [17] for the detection of HSV, VZV, CMV, rubella virus, chikungunya virus, *Toxoplasma*, and *Mycobacterium tuberculosis*. Controls (from 25 cases) included patients undergoing routine cataract surgery and from patients with an established diagnosis of Vogt-Koyanagi Harada disease. The microbiologist performing the analysis was masked to the diagnosis.

SES briefly involved extraction of DNA and RNA from the aqueous. RNA was converted to cDNA using multiplex specific primers of rubella virus and chikungunya virus. DNA and cDNA were then multiplex amplified for all the organisms simultaneously. The amplicons were identified by hybridization on a proprietary platform embedded with target sequences of the signature genes for all seven pathogens. Primers and targets for HSV, CMV, VZV, *Toxoplasma*, and MTb are already described in the literature [17]. Three genes in the case of HSV and CMV and two genes in the case of VZV per organism were amplified for identification, while single genes were targeted for *Toxoplasma* and MTb.

An informed consent was taken from all the participants in this study and this study was approved by our institutional review board. The results were statistically analyzed using the SPSS version 15 (SPSS Inc., Chicago, IL, USA).

## Competing interests

The authors declare that they have no competing interests.

## Authors’ contributions

KB planned the design of the study, collection and interpretation of data, and overall compilation of the data, and wrote the manuscript. MA carried out the collection of data. SRG and BVRK carried out the molecular diagnostic studies. KRM was involved in the collection of data, performing the procedure, and overall supervision of the manuscript. All authors read and approved the final manuscript.
